# Evaluation of salivary parameters and *Streptococcus*’ *Mutans* count in children with cerebral palsy in Egypt: a case control study

**DOI:** 10.1186/s12903-022-02447-0

**Published:** 2022-09-19

**Authors:** Sara M. Quritum, Amel M. Ali, May M. Raouf, Tarek E. I. Omar, Karin M. L. Dowidar

**Affiliations:** 1 Department of Pediatric Dentistry and Dental Public Health, Faculty of Dentistry, Alexandria University, Alexandria, Egypt; 2 Department of Medical Microbiology and Immunology, Faculty of Medicine, Alexandria University, Alexandria, Egypt; 3 Department of Pediatrics, Faculty of Medicine, Alexandria University, Alexandria, Egypt

**Keywords:** Cerebral palsy, Caries experience, Salivary parameters, Salivary total antioxidant, MALDITOF, *Streptococcus mutans*

## Abstract

**Background:**

Children with cerebral palsy (CP) are at high risk for dental caries. Alteration of some salivary properties encountered among them compared to healthy children, could play a role in this elevated risk.

**Objectives:**

The aim of the present study was to assess salivary physicochemical properties; including total antioxidant (TAC), flow rate, viscosity, pH and buffering capacity, as well as *Streptococcus mutans* level among children with CP, also to correlate these variables to their caries experience.

**Materials and methods:**

This case control study included 80 children with CP, study group (SG) and matched number of healthy children for control group (CG). Interview-based questionnaire, clinical examination, salivary biochemical and microbiological investigations using MALDI-TOF were done.

**Results:**

In SG, the caries experience in primary teeth dmft and *S. mutans* log value were significantly higher than CG (*P* = 0.039, *P* = 0.002) while unstimulated salivary flow rate, buffering capacity and salivary TAC were significantly lower (*P* < 0.0001). Multivariate linear regression showed that the presence of CP was significantly associated with the greatest variation in caries experience in the primary teeth and permanent teeth. Higher unstimulated salivary flow rate, or an increase in buffering capacity by 1 ml of acid/ml of saliva were associated with lower number of the affected primary and permanent teeth. On the other hand, One-unit increase in *S. mutans* log count and higher salivary TAC were associated with higher caries experience.

**Conclusion:**

Children with CP have higher caries experience (dmf) due to lower salivary protective factors and higher *S. mutans* counts*.*

**Supplementary Information:**

The online version contains supplementary material available at 10.1186/s12903-022-02447-0.

## Introduction

Cerebral palsy (CP) is a group of permanent muscle tone disorders due to non-progressive brain injury during fetal or infant brain development [1]. Prevalence estimates range from 1.5 to 3/1000 in western countries, with much higher and wider range, 2–10/1000 live births, in the developing areas. Prevalence in Egypt was estimated to be 2.04 per 1000 live births [2].

The group of Surveillance of Cerebral Palsy in Europe (SCPE) had classified CP into three main groups according to the type of abnormal resting muscle tone motion and topographical distribution. This include spastic, ataxic or dyskinetic Spastic is the most common type, accounting for nearly 70–80% of all CP cases [3]. Mental and motor disability-related factors among these children could interfere with achievement of normal daily activities including oral health and predisposing to dental problems. Moreover, alteration of salivary physiochemical properties observed in CP children play a role in their elevated caries risk [4].

Saliva has multifunctional roles in the oral cavity; it plays a critical role in maintaining oral homeostasis. Total Antioxidant Capacity (TAC) of saliva might be related directly to caries risk especially in children; suggesting that it can be used as an indicator evaluating the individuals’ dental caries activity. Moreover, it was noted that pH and buffering capacity of saliva decreases with caries activity, thus these factors play an important role in development of caries [5].

Previous studies showed that the base-line levels of salivary *S. mutans* are correlated positively with both past caries experience (DMFS index) and with 1-year caries increment [6]. In recent years, Matrix Assisted Laser Desorption Ionization Time-Of-Flight mass spectrometry (MALDI-TOF MS) had replaced conventional bacterial identification methods. It is a rapid, reliable and consumable cost-effective method for identification of bacteria and fungi that was introduced in clinical microbiology laboratories since early 2000s. It detects unique microbial ribosomal proteins which is then compared to fingerprint libraries leading to pattern matching. It has been FDA approved after comparing its results to16s rRNA sequencing [7].

Studies carried out regarding salivary parameters and its relation to dental caries among individuals with CP, particularly the total antioxidant capacity, are few. The objective of the present case control study was to clinically assess the sialometric and sialochemical alterations, including flow rate, viscosity, pH, buffering capacity and TAC, as well as the *Streptococcus mutans* levels, and their relation to dental caries among children with CP and healthy children. The alternative hypothesis of the current study was that the salivary physicochemical and microbiological parameters would differ between children who have cerebral palsy or healthy children.

## Subjects and methods

Ethical approval was obtained from the Research Ethics Committee, Faculty of Dentistry, Alexandria University, Egypt (IRB 00010556–IORG 0008839) and the study was performed in full accordance with the Helsinki declaration. A written informed consent was obtained from parents/guardians for examination and publication, after thorough explanation of the study methodology and aims as well as benefits and risks.

### Studied population

This is a case–control study conducted in Alexandria, Egypt, over 18 months from 2018 to 2020. Children with CP were recruited from Alexandria University Children’s Hospital (AUCH) and its outpatient-clinics (Pediatric Neurology and Physical Medicine), from Pediatric Dental Clinic in the Department of Pediatric Dentistry and Dental Public Health, Faculty of Dentistry, Alexandria University, and from governmental institutions for children with special needs. The study population consisted of two groups: children with CP, in the study group (SG) and healthy children in the control group (CG). Both groups were matching concerning age, sex, health district and socioeconomic level.

### Sample size estimation

The assumptions made for sample size estimation was based on mean value of the salivary total antioxidant capacity (S-TAC) in CP and healthy population and from secondary analysis from a previous study [8, 9]. The power of the study was 80% and alpha error was 5% and beta error = 20%.

Eighty subjects were enrolled into each subgroup in the age range of 3 to 11 with total, 160 children. A neurologist had previously diagnosed children with CP. Those who had any other medically compromising condition, severely uncooperative, or did not get parental consent were excluded from the study. In addition, children taking antibiotics or medication that could affect salivary secretion were not included.

### Questionnaire

Data were collected from parent’s/caregivers using an interview-based questionnaire, that assessed socio-demographic profile (child’s age and sex, parental education, and occupation), previous dental visits, and oral hygiene behaviors (brushing, frequency of brushing, use of fluoridated toothpaste, parental supervision of brushing), and dietary habits (frequency of sugary snacks daily, food consistency) [10] (Additional file 1).

### Clinical examination

Children were examined to assess caries experience, following the World Health Organization (WHO) criteria, using the dmft index for primary dentition, and dft and DMFT indices for mixed dentition [11]. Training and calibration for dental caries assessment was done (Kappa value for caries diagnosis was 0.9) [12]. Oral hygiene status was assessed using the Simplified Oral Hygiene Index (OHI-S) [13].

#### Salivary collection and assessment [14]

Unstimulated whole saliva sample was collected in a morning appointment using a portable suctioning device. Instructions were given to stop eating, drinking, or brushing for 2 h before collection. The salivary flow rate was assessed [14] (ml/min), as well as salivary viscosity [14] and drooling.

### Biochemical analysis [5]

In the biochemistry lab, the salivary pH was measured using a digital portable pH meter, while the buffering capacity was determined by titration of 0.1 ml of 0.01 N HCl solution. One milliliter of the sample was centrifuged at 3000 rpm for 5 min, to remove bacteria and cellular debris. The clear supernatant fluid was kept frozen at − ve 80 degrees centigrade until the execution of the TAC analysis. This was analyzed using the antioxidant colorimetric assay kit and a spectrophotometer; to evaluate optical absorption of the sample.

### Microbiological assessment [15]

Freshly prepared pre-reduced transport media (Thioglycollate broth) was used. The salivary samples were vortexed and diluted twice 1:10x and 1:100x. Mitis Salivarius Bacitracin (MSB) agar was prepared according to the manufacturer’s instructions. The agar plates were divided into 4 sectors. Using an inoculation loop, 1 μl of both vortexed diluted samples were streaked onto the specified sector on each plate respectively. The plates were then incubated in anaerobic jar for 48 h at 37 °C. *S. mutans* colonies were provisionally identified based on colonial morphology, Gram staining and catalase.

Matrix Assisted Laser Desorption and Ionization Time of Flight Mass Spectrometry “MALDI-TOF MS” ultraflex (Brüker Daltonik, Germany) was used to confirm bacterial identification using protein extraction method [16]. Briefly, MALDI-TOF MS analysis was operated in the positive linear mode ranging from 2000 to 20,000 m/z. The generated Spectra were compared to fingerprint database by using the Bruker Biotyper 3.1 software.

Colonies identified of being *S. mutans* were counted through a *Semi quantitative method*, using a magnifying glass. Number of colonies for 1 ml of saliva (CFU/ml) = actual colony count × dilution factor (10 or 100) × the cultured volume.

### Statistical analysis

Statistical analysis was carried out using statistical package for social sciences (SPSS for windows, version 23.0, Inc. Chicago, IL, USA). Previous dental visits, oral hygiene practices and dietary habits were compared between the 2 groups using chi-squared test (or Fisher exact test as indicated). Oral hygiene index scores were compared using Mann–Whiney U-test. Linear regression models were done to determine which independent variables were significant for explaining the variation in the caries experience in primary and permanent teeth (dependent variable). Regression coefficient, 95% confidence intervals and estimates of effect size were calculated.

## Results

**Fig. 1:**
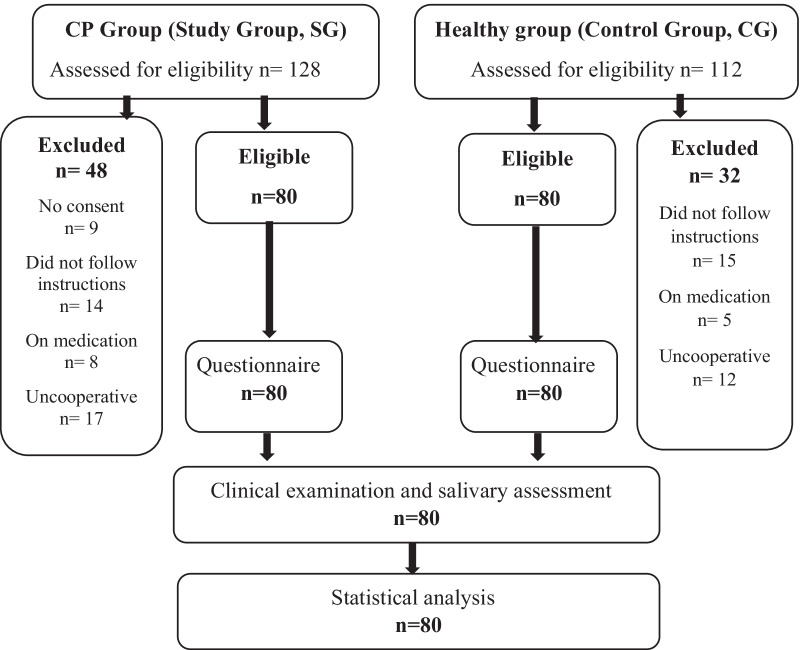
Flow chart of study participants

Total of 249 children ranging from 3 to 11 years of age, were initially examined during the period from 2019 until 2020. Eighty children were enrolled and allocated in the main 2 groups, according to their systemic condition (Fig. 1).

The mean age of SG in the study was 6.75 ± 2.62 years; about two thirds of them were males. Both groups were comparable as regards age and sex (*P* = 1 and 0.8 respectively). As reported by parents in the CP group, they were significantly less likely to visit the dentist last year than healthy children (*P* < 0.0001). Children who visited the dentists in both groups were mainly suffering from dental pain.

**Fig. 2:**
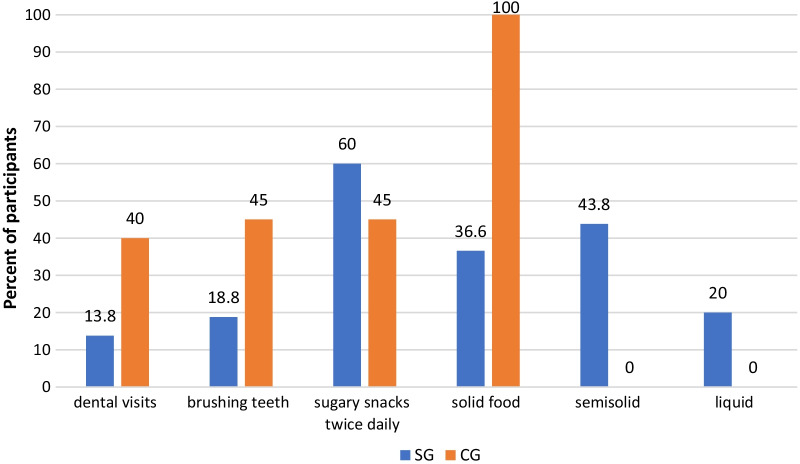
Oral health practices among cerebral palsy and healthy children

Healthy children were noticed to brush their teeth more than children did in SG (*P* < 0.0001). Most of children with CP were supervised by their parents during tooth brushing, however the majority of them reported problems upon brushing (*P* < 0.0001). As regards snacking habits, sugary snacks were consumed twice daily or more by children with CP more frequently than healthy children (60%, *P* < 0.001). Additionally, higher percentage of children in SG are eating semisolid food (43.8%), whereas all healthy children were on solid diet (Fig. 2).

### Comparison of caries experience and oral hygiene status between CP and healthy children

The mean value of OHI-S in the CP (2.14 ± 0.68) was significantly higher than that of healthy children (1.32 ± 0.56), (*P* < 0.0001). Children with CP have significantly higher dmft (6.86 ± 7.11) than healthy children (5.16 ± 5.21), (*P* = 0.039). They have significantly more decayed (6.44 ± 6.64) primary teeth (*P* < 0.0001) as well as fewer missing (0.28 ± 0.67) and filled (0.14 ± 0.47) primary teeth (*P* = 0.04, 0.01 respectively). On the other hand, DMFT did not differ significantly between CP and healthy children (*P* = 0.52) (Table 1).

**Table 1:** Caries experience and oral hygiene among cerebral palsy and healthy children

	SG (n = 80)	CG (n = 80)	*P* of MWU test
OHI-S^§^			
Mean ± SD	2.14 ± 0.68	1.32 ± 0.56	< 0.0001*
Median (range)	2.0 (1.1–3.4)	1.3 (0.3–2.5)	
Primary teeth			
Decay (d)			
Mean ± SD	6.44 ± 6.64	3.79 ± 3.83	< 0.0001*
Median (range)	6 (0–11)	2 (0–7)	
Missing (m)			
Mean ± SD	0.28 ± 0.67	0.68 ± 0.82	0.041*
Median (range)	0 (0–2)	0 (0–2)	
Filled (f)			
Mean ± SD	0.14 ± 0.47	0.68 ± 1.25	0.011*
Median (range)	0 (0–2)	0 (0–4)	
Caries experience (dmft)			
Mean ± SD	6.86 ± 7.11	5.16 ± 5.21	0.039*
Median (range)	7 (0–12)	4 (0–9)	
Permanent teeth			
Decay (D)			
Mean ± SD	1.18 ± 1.56	0.67 ± 0.97	0.42
Median (range)	0 (0–4)	0 (0–3)	
Missing (M)			
Mean ± SD	0.14 ± 0.47	0.05 ± 0.22	0.56
Median (range)	0 (0–2)	0 (0–1)	
Filled (F)			
Mean ± SD	0	0.19 ± 0.51	0.07
Median (range)	0	0 (0–2)	
Caries experience (DMFT)			
Mean ± SD	1.32 ± 1.73	0.90 ± 1.26	0.52
Median (range)	0 (0–4)	0 (0–4)	

SG: Study group = children with cerebral palsy

CG: control group = health children

MWU test: Mann Whitney test

*Statistically significant at *P* < 0.05

^§^Student T test used

### Comparison of salivary parameters between CP and healthy children

Statistically significant lower unstimulated salivary flow rate was found among CP (0.34 ± 0.12) as compared to the healthy children (0.54 ± 0.18, (*P* < 0.0001). Additionally, CP also recorded significantly lower initial pH (7.05 ± 0.53) and buffering capacity (0.62 ± 0.16) than healthy children (7.63 ± 0.44 and 0.75 ± 0.17), (*P* < 0.0001 for both). CP demonstrated significantly lower salivary TAC (2.65 ± 1.10) than healthy children (3.29 ± 1.05), (*P* < 0.0001) and higher *S. mutans* log value in the CP (6.75 ± 0.25) than the healthy children (6.63 ± 0.23), (*P* = 0.002). The consistency of saliva did not differ significantly between the 2 groups (*P* = 0.053). However, a difference existed between them as regards drooling, since 46.6% of CP were suffering from drooling, while no one in the CG had this condition (*P* < 0.0001) (Table 2).

**Table 2:** Comparison of salivary parameters between cerebral palsy and healthy children

Salivary parameters	SG (n = 80)	CG (n = 80)	*P* value
Unstimulated flow rate (ml/min)^§^			
Mean ± SD	0.34 ± 0.12	0.54 ± 0.18	< 0.0001*
Median (range)	0.30 (0.10–0.60)	0.50 (0.20–1.00)	
Initial PH^§^			
Mean ± SD	7.05 ± 0.53	7.63 ± 0.44	< 0.0001*
Median (range)	7.31 (5.91–7.57)	7.66 (6.70–8.32)	
Buffering capacity^§^			
Mean ± SD	0.62 ± 0.16	0.75 ± 0.17	< 0.0001*
Median (range)	0.58 (0.20–0.90)	0.80 (0.35–1.20)	
Salivary TAC (mmol/L)^§^			
Mean ± SD	2.65 ± 1.10	3.29 ± 1.05	< 0.0001*
Median (range)	2.43 (0.70–4.90)	3.36 (0.90–4.90)	
*S. mutans* (log count)^§^			
Mean ± SD	6.75 ± 0.25	6.63 ± 0.23	0.002*
Median (range)	6.75 (6.15–7.38)	6.64 (6.10–7.10)	
Saliva consistency			
Watery & clear: no (%)	52 (65%)	63 (78.8%)	0.053
Thick or sticky: no (%)	28 (35%)	17 (21.3%)	
Drooling^†^			
No: no (%)	43 (53.8%)	80 (100%)	< 0.0001*
Yes: no (%)	37 (46.3%)	0	

SG: Study group = children with cerebral palsy

CG: control group = health children

*Statistically significant at *P* < 0.05

^¶^Chi-squared test used

^†^Fisher's Exact test used

^§^T-test used

The association between caries experience in primary teeth (dmft) and the independent variables was represented in the linear regression model in Table 3. Multivariate linear regression showed that the presence of CP was significantly associated with the greatest variation in caries experience in the primary teeth (partial eta square = 0.43), followed by the level of salivary *S. mutans* (partial eta square = 0.35) and unstimulated salivary flow rate (partial eta square = 0.31). Children with CP had higher caries experience in their primary teeth by about 6 teeth more than healthy children (regression coefficient = 5.87, 95% CI = 4.95, 6.78). Higher dmft score by about one tooth was also observed in participants with previous visit to the dentist, as well as those children consuming liquid diet than those eating solid food. However, children who brushed their teeth had lower dmft score than those who did not brush. One ml increase in the flow rate per minute was associated with lower caries experience in the primary teeth by about 4 teeth (regression coefficient =  − 4.46, 95% CI =  − 5.55, − 3.36). Higher salivary buffering capacity by about 1 ml of acid/ml of saliva was associated with lower dmft by about 3 teeth (regression coefficient =  − 3.45, 95% CI =  − 4.39, − 2.51). Similarly, watery saliva was associated with lower values of dmft scores compared to thick saliva (regression coefficient =  − 0.79, 95% CI =  − 1.23, − 0.34). On the other hand, an increase in salivary TAC by 1 mmol/L or one-unit increase in log count of *S. mutans* were associated with higher caries experience in the primary teeth by about 4 teeth (regression coefficient of salivary TAC = 3.95, 95% CI = 3.56, 4.33, while for *S. mutans* log value = 4.47, 95% CI = 2.66, 6.27). The overall model represents high model fit (*P* < 0.0001, Adjusted R2 = 0.93).

**Table 3:** The association of independent variables (SES, oral health practices, and salivary parameters) and caries experience in primary teeth (dmft) in the entire sample

	Univariate regression			Multivariate regression		
	*P* value	Unadjusted regression coefficient (95% C.I.)	Partial eta squared	*P* value	Adjusted regression coefficient (95% C.I.)	Partial eta squared
CP versus normal	0.013*	1.35 (0.29, 2.41)	0.08	< 0.0001*	5.87 (4.95, 6.78)	0.43
Age	0.51	0.07 (− 0.14, 0.28)	0.005			
Male versus female	0.81	0.15 (− 1.03, 1.32)	0.001			
Illiterate father versus university educated	0.09	1.49 (− 0.21, 3.2)	0.02			
Father occupation: Unemployed versus employed	0.38	0.61 (− 0.76, 1.98)	0.001			
Illiterate mother versus university educated	0.37	0.98 (− 1.18, 3.13)	0.005			
Mother occupation unemployed versus employed	0.17	0.91 (− 0.39, 2.21)	0.01			
Visiting dentist	< 0.0001*	2.87 (1.7, 4.04)	0.13	< 0.0001*	0.83 (0.44, 1.22)	0.11
Tooth-brushing	0.004*	− 1.71 (− 2.87, − 0.55)	0.05	0.001*	− 0.70 (− 1.10, − 0.29)	0.07
Sugary snacks twice daily	< 0.0001*	3.22 (2.23, 4.22)	0.21	0.15	0.30 (− 0.11, 0.7)	0.01
Eating semiliquid diet versus solid	0.053	1.71 (− 0.01, 2.36)	0.02			
Eating liquid diet versus solid	< 0.0001*	6.02 (4.39, 7.65)	0.25	0.002*	1.24 (0.47, 2.0)	0.07
Unstimulated flow rate	< 0.0001*	− 6.61 (− 9.69, − 3.53)	0.1	< 0.0001*	− 4.46 (− 5.55, − 3.36)	0.31
Initial pH	0.001*	− 4.49 (− 7.22, − 1.76)	0.06	0.61	− 0.26 (− 1.28, 0.76)	0.002
Buffering capacity	< 0.0001*	− 5.0 (− 6.15, − 3.85)	0.32	< 0.0001*	− 3.45 (− 4.39, − 2.51)	0.27
Salivary total antioxidant capacity	< 0.0001*	1.49 (0.98, 1.99)	0.18	< 0.0001*	3.95 (3.56, 4.33)	0.24
*S. mutans* (log count)	< 0.0001*	8.15 (6.29, 10.0)	0.33	< 0.0001*	4.47 (2.66, 6.27)	0.35
Saliva viscosity: watery versus thick	< 0.0001*	− 4.15 (− 5.2, − 3.09)	0.28	0.001*	− 0.79 (− 1.23, − 0.34)	0.08

F = 166.42, *p* < 0.0001*, adjusted R^2^ = 0.93, *statistically significant at *P* < 0.05

Linear regression model assessing the association between caries experience in permanent teeth (DMF) and the independent variables was described in Table 4. The presence of CP was associated with higher DMFT by about 4 teeth (regression coefficient = 3.79, 95% CI = 2.66, 4.92). Children who brushed their teeth had less caries experience in permanent teeth by about 2 teeth than children who did not brush (regression coefficient =  − 1.79, 95% CI =  − 2.29, − 1.29). While children consuming liquid diet had significantly higher DMFT than those eating solid food (regression coefficient = 2.62, 95% CI = 1.29, 3.95). Higher unstimulated salivary flow rate by 1 ml/min was associated with lower number of the affected permanent teeth by about 5 teeth (regression coefficient =  − 5.08, 95% CI =  − 6.43, − 3.72). Similarly, an increase in buffering capacity by 1 ml of acid/ml of saliva was associated with lower DMFT (regression coefficient =  − 1.69, 95% CI =  − 2.98, − 0.40). On the other hand, One-unit increase in *S. mutans* log count was associated with higher caries experience in permanent teeth by about 3 teeth (regression coefficient = 3.16, 95% CI = 2.68, 3.63). Whereas, higher salivary TAC by 1 mmol/L was associated with an increase in DMFT score by about one tooth (regression coefficient = 0.96, 95% CI = 0.47, 1.44). The overall model explained high amount of the variation in caries experience in permanent teeth (*P* < 0.0001, adjusted R2 = 0.86).

**Table 4:** The association of independent variables (SES, oral health practices and salivary parameters) and caries experience in permanent teeth (DMFT) in the entire sample

	Univariate regression			Multivariate regression		
	*P* value	Unadjusted regression coefficient (95% C.I.)	Partial eta squared	*P* value	Adjusted regression coefficient (95% C.I.)	Partial eta squared
CP versus normal	0.017*	1.21 (0.22, 2.20)	0.04	< 0.0001*	3.79 (2.66, 4.92)	0.23
Age	0.27	− 0.11 (− 0.31, 0.09)	0.008			
Male versus female	0.93	0.02 (− 0.51, 0.55)	0.001			
Illiterate father versus university educated	0.051	0.77 (− 0.04, 1.54)	0.04			
Father occupation: unemployed versus employed	0.93	0.03 (− 0.66, 0.73)	0.001			
Illiterate mother versus university educated	0.32	0.43 (− 0.43, 1,30)	0.012			
Mother occupation unemployed versus employed	0.47	0.21 (− 0.67, 0.79)	0.006			
Visiting dentist	0.08	0.48 (− 0.07, 1.03)	0.13			
Tooth-brushing	0.024*	− 0.61 (− 1.13, − 0.08)	0.06	0.002*	− 1.79 (− 2.29, − 1.29)	0.12
Sugary snacks twice daily	0.019*	0.63 (0.11, 1.15)	0.06	0.51	0.16 (− 0.33, 0.65)	0.006
Eating semiliquid diet versus solid	0.52	0.38 (− 0.79, 1.55)	0.005			
Eating liquid diet versus solid	0.046*	1.28 (0.02, 2.54)	0.046	< 0.0001*	2.62 (1.29, 3.95)	0.17
Unstimulated flow rate	< 0.0001*	− 7.04 (− 9.84, − 4.24)	0.14	< 0.0001*	− 5.08 (− 6.43, − 3.72)	0.27
Initial pH	0.15	− 0.95 (− 2.26, 0.35)	0.02	0.54	− 0.33 (− 1.67, 1.00)	0.003
Buffering capacity	0.001*	− 0.99 (− 1.59, − 0.40)	0.11	0.011*	− 1.69 (− 2.98, − 0.40)	0.08
Salivary total antioxidant capacity	0.028*	0.28 (0.03, 0.52)	0.06	< 0.0001*	0.96 (0.47, 1.44)	0.17
*S. mutans* (log count)	< 0.0001*	2.05 (1.05, 3.05)	0.16	< 0.0001*	3.16 (2.68, 3.63)	0.20
Saliva viscosity: watery versus thick	0.001*	− 0.92 (− 1.44, − 0.41)	0.13	0.59	− 0.16 (− 0.74, 0.42)	.004

F = 97.35, *p* < 0.0001*, Adjusted R^2^ = 0.86, *Statistically significant at *P* < 0.05

**Fig. 3:**
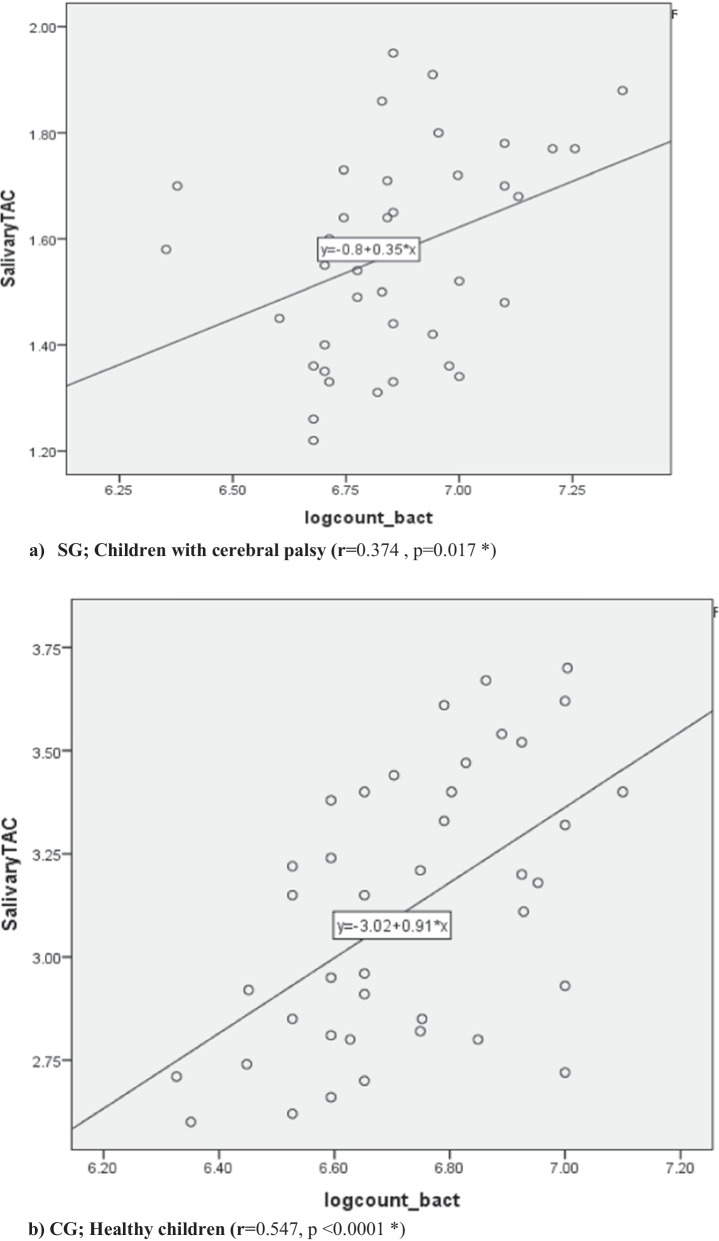
Correlation between salivary total antioxidant capacity and the level of *S. mutans* among **a** SG; children with cerebral palsy, **b** CG; healthy children

Significant moderate positive correlation was observed between TAC and the level of *S. mutans* in the saliva of SG as well as CG as represented in Fig. 3.

## Discussion

Dental caries remains a widely prevalent disease despite tremendous advances in prevention and treatment [17]. The interplay of multiple factors was found to influence caries development and progression. Several in vitro studies have discussed the biological plausibility that changes in salivary parameters can contribute to the development of dental caries [5, 18]. However, controversy exists about the incidence of dental caries and its associated salivary risk factors in children with CP [19]. Identification of individuals at high risk of caries as children with special health care needs would be of considerable importance in allocating resources for caries prevention.

The relatively wide age range in the present study allows the evaluation of different risk factors for dental caries affecting primary and mixed dentitions. The results of the current research accept the proposed hypothesis as intra-oral examination revealed that SG had higher caries experience in their primary and permanent teeth than CG. However, this difference was only statistically significant in the primary teeth (dmft). Similar findings were found by Jaber et al. [20]. Ruiz et al. also reported no significant difference in caries experience in permanent teeth (DMF) between both groups among elder children [4]. On the other hand, Grzic et al. reported no significant difference in a tooth morbidity (DMTF/dft) between the SG and CG [21].

Multivariable linear regression in our results clearly shows that the presence of CP was significantly associated with great variation in caries experience in both primary and permanent teeth. Additionally, children with CP have significantly more decayed primary teeth than healthy children, representing the major contribution of the dmft score. This was in line with former studies reporting higher treatment needs and less dental services provided for CP children [20, 22].

On the other hand, lower caries experience that was observed in the permanent than primary teeth among both CP and healthy children, might probably be due to the fewer number of erupted permanent teeth, and less exposure time to the cariogenic oral environment than primary teeth [23]. Moreover, the physical abilities of CP children could change with time [24], that may result in more self-dependence in their daily activities, as well as changing the dietary consistency to a more solid food, and hence better oral hygiene as they grow up [25].

The level of oral hygiene status reflects the efficiency and frequency of brushing and possibly of dietary habits. In line with previous reports, the current research reported unsatisfactory oral hygiene in SG [26]. This could be attributed to impaired natural cleansing by the oral musculature, reduced manual dexterity, some degree of cognitive impairment, which further hinders the adoption of adequate oral hygiene practices and result in partial or total reliance on the caregiver [27]. Quritum et al. reported poorer oral hygiene within children with CP who have dental caries compared to caries free CP children when studying the impact of oral hygiene practices and dietary habits on their caries experience [9].

It has been long recognized that saliva acts as a mirror for body's health and can be used as a non-invasive diagnostic tool for monitoring general or oral conditions in children and non-cooperative subjects [28]. Alteration of salivary parameters encountered in children with CP compared to their healthy counterparts, could play a pivotal role in caries incidence and progression [29].

Unfortunately, few studies have been published on the salivary composition of children with CP. Unstimulated whole saliva was considered in the present study as it correlates to clinical conditions more accurately than stimulated saliva [14]. Children with CP had demonstrated about 40% reduction in their salivary flow rate, which may explain the higher caries experience observed among them. Parallel findings were observed by Diniz et al. [30]. This may be due to their complete dependence on their caregivers to offer them liquids, their persistent pathological oral reflexes can also interfere with the normal oral function and result in impaired level of hydration [31]. On the other hand, Tahmassebi and Curzon found no difference in the flow rate between SG and CG. However, their study was based on a small number of participants and used different methods of salivary collection [32].

In line with the literature, a high prevalence of drooling was observed among children with CP, despite the lower values of salivary flow rates recorded among them [33]. Tahmassebi reported that drooling among these children is not due to hyper salivation, but it usually results from incontinency secondary to impaired cerebral control of orofacial musculature. Other predisposing factors could also include quadriplegic topographical pattern, absence of cervical control, epilepsy, intellectual disability, lack of speech, open anterior bite, as well as dysphasia [32].

The consistency of the saliva can affect its capacity to flush microorganisms and substrates, as well as maintaining the oral cleanliness. In the present study, it was observed that greater proportion of SG have thick and sticky saliva than CG but this difference was not found to be statistically significant. This could be referred to reduced water content and increase protein content in the saliva of CP children [34].

Salivary pH and the buffering capacity are essential parameters in controlling the ion exchanges during re-mineralization and demineralization of enamel. They are determined by the hydrogen bicarbonate balance in saliva. It was noticeable that SG had statistically significant lower salivary pH and buffering capacity than CG (*P* < 0.0001 for both). These findings were in synchronous with previous research on CP children done by Subramaniam et al. [35]. This can result in an increased susceptibility to demineralization and caries intiation. On the other hand, Tahmassebi reported no significant difference [32]. Linear regression showed that an increase in the salivary buffering capacity was significantly associated with lower caries experience in both primary and permanent teeth. However, this association was not evident with salivary pH, as it is a labile parameter highly influenced by the type and timing of food intake as well as the individual’s oral hygiene habits. Whereas, buffering capacity represents innate resistance to neutralize acids, thus it is more efficient in predicting caries experience.

Total antioxidant capacity (TAC) was estimated in the present study, as it was suggested that free radical/ reactive oxygen species and antioxidant systems appear to act in concert rather than alone. Furthermore, not all the compounds with antioxidant properties are believed to be identified [36]. Comparing the level of salivary TAC among CP and healthy children demonstrated a statistically significant lower level among SG. The efficacy of total antioxidant system may be related to many factors such as antioxidants potency and level, amount of free radical production, individual genetic basis, dietary intake, physical activity, hormones, and stress.

It was reported that children with CP suffer from high levels of oxidative stress throughout their lifespan, consequent to vitamin deficiency, malnutrition, environmental factors as well as epileptic seizures. That is why the radical-scavenging antioxidants get consumed by the increased free radical activity associated with this condition [37]. These finding is partially in agreement to a previous study done by Subramaniam et al. [8], where an inverse relation between TAC and dental caries was reported. This could be attributed to difference in dmft values, dietary pattern, oral hygiene practice and genetics. Additionally, linear regression models clearly represented that salivary level of TAC was significantly associated with the number of affected primary and permanent teeth.

Bacteria produce free radicals during dental decay progression and their number appears to vary directly with caries activity [38]. Thus, increased level of salivary TAC can be a *compensatory mechanism*, to neutralize the effect of the high oxidant level. This could also be verified by the statistically significant positive moderate correlation observed between the level of TAC and *S. mutans* in the current study.

Perhaps in the near future, through longitudinal studies, a TAC index will be introduced as a marker of caries susceptibility in children.

Microbiological examination of the saliva was done through anaerobic culturing of *S. mutans*, the main organism responsible for caries initiation [39]. its presence was confirmed using MALDI-TOF MS, as it represents a rapid and accurate proteomic approach for identification of bacteria, compared to the conventional biochemical and metabolic techniques [40].

Significantly, higher level of *S. mutans* was found in the saliva of children in SG. Parallel finding was reported by Santos et al. [41]. This could be attributed to poorer oral hygiene and faulty dietary habits and reduced self-cleansing mechanism by the impaired oro-motor function, resulting in more food debris. Such practices promotes the growth of these cariogenic microorganisms.

Brain damage associated with CP can be responsible for the alteration in the salivary parameters, which could explain the higher caries experience encountered in this group [42]. The management of children with CP should be done through a multi-disciplinary team approach, involving the cooperation of a wide array of specialties including pediatric dentistry. The presence of multiple risk factors for dental caries in children with CP necessitates vigorous preventive advice and high quality of dental care. Preventive measures must be introduced for children with CP as soon as their condition is diagnosed to improve the general and oral health status.

## Limitations

The study had some challenges and limitations. Dental caries is a multi-factorial disease, and it is impossible to investigate all factors in one study. Additionally, case control studies do not allow the establishment of causal relationships. The potential for recall bias is a threat. It was not also possible to strictly match the nutritional program of children under the study the day before sampling.

## Conclusion

Compared with the healthy group, caries experience in primary teeth dmft and *S. mutans* log value were significantly higher in CP patients, while unstimulated salivary flow rate, buffering capacity and salivary TAC were significantly lower. This alteration in physicochemical and biological properties of saliva among children with CP support the possibility of salivary gland impairment and could explain their higher caries experience.

### What does this study add to the literature?

- Alteration in salivary protective factors among children with CP; such as lower levels of unstimulated salivary flow rate, buffering capacity and salivary TAC and higher levels of *S. mutans* log value and salivary TAC, would make them at higher risk for having dental caries.
- A positive correlation exists between the level of TAC and *S. mutans* counts.

## Supplementary Information

**Additional file 1.** Questionnaire.

## Data Availability

The datasets used and/or analyzed during the current study are available from the corresponding author on reasonable request.

## Abbreviations

CP: Cerebral palsy
SG: Study group
CG: Control group
SES: Socioeconomic status
TAC: Total antioxidant capacity
MALDI-TOF MS: Matrix Assisted Laser Desorption Ionization Time-Of-Flight mass spectrometry
dmft: Primary caries index
DMFt: Permanent caries index

## References

[1] Graham HK, Rosenbaum P, Paneth N (2016). Cerebral palsy. Nat Rev Dis Prim.

[2] Abas O, Abdelaziem F, Kilany A (2017). Clinical spectrum of cerebral palsy and associated disability in South Egypt: a local survey study. Open Access Maced J Med Sci.

[3] Surveillance of cerebral palsy in Europe: a collaboration of cerebral palsy surveys and registers. Surveillance of Cerebral Palsy in Europe (SCPE). Dev Med Child Neurol. 2000;42(12):816–24. 10.1017/s0012162200001511 11132255

[4] Ruiz LA, Diniz MB, Loyola-Rodriguez JP, Habibe CH, Garrubbo CC, Santos MTBR (2018). A controlled study comparing salivary osmolality, caries experience and caries risk in patients with cerebral palsy. Med Oral Patol Oral y Cir Bucal.

[5] Muchandi S, Walimbe H, Bijle M, Nankar M, Chaturvedi S, Karekar P (2015). Comparative evaluation and correlation of salivary total antioxidant capacity and salivary pH in caries-free and severe early childhood caries children. J Contemp Dent Pract.

[6] Neves AB, Lobo LA, Pinto KC (2015). Comparison between clinical aspects and salivary microbial profile of children with and without early childhood caries: a preliminary study. J Clin Pediatr Dent.

[7] Li Y, Shan M, Zhu Z (2019). Application of MALDI-TOF MS to rapid identification of anaerobic bacteria. BMC Infect Dis.

[8] Subramaniam P, Mohan Das L, Babu KL (2014). Assessment of salivary total antioxidant levels and oral health status in children with cerebral palsy. J Clin Pediatr Dent.

[9] Quritum S, Dowidar K, Ahmed A, Omar T (2019). Impact of oral health behaviours on dental caries in children with cerebral palsy: a case-control study. Alex Dent J.

[10] El Khatib AA, El Tekeya MM, El Tantawi MA, Omar T (2014). Oral health status and behaviours of children with autism spectrum disorder: a case-control study. Int J Paediatr Dent.

[11] World Health Organization (2013). Oral health surveys: basic methods.

[12] McHugh ML (2012). Interrater reliability: the kappa statistic. Biochem Med.

[13] Greene JC, Vermillion JR (1964). The simplified oral hygiene index. J Am Dent Assoc.

[14] Nunes LAS, Mussavira S, Bindhu OS (2015). Clinical and diagnostic utility of saliva as a non-invasive diagnostic fluid: a systematic review. Biochem Med.

[15] Tille P, Tille P (2017). Traditional cultivation and identification. Bailey and Scotts’s diagnostic microbiology.

[16] Abouseada N, Raouf M, El-Attar E, Moez P (2017). Matrix-assisted laser desorption ionisation time-of-flight mass spectrometry rapid detection of carbapenamase activity in *Acinetobacter baumannii* isolates. Indian J Med Microbiol.

[17] American Academy on Pediatric Dentistry Council on Clinical Affairs (2008). Policy on use of a caries-risk assessment tool (CAT) for infants, children, and adolescents. Pediatr Dent.

[18] Cunha-Cruz J, Scott J, Rothen M, Mancl L, Lawhorn T, Brossel K (2013). Salivary characteristics and dental caries: evidence from general dental practices. J Am Dent Assoc.

[19] Marwaha M, Bansal K, Sehrawat N, Chopra R (2014). Cerebral palsy: a dental update. Int J Clin Pediatr Dent.

[20] Jaber MA, Allouch T (2014). Dentofacial abnormalities and oral health status in children with cerebral palsy. JBR J Interdiscip Med Dent Sci 2015.

[21] Grzic R, Bakarcic D, Prpic I, Jokic NI, Sasso A, Kovac Z (2011). Dental health and dental care in children with cerebral palsy. Coll Antropol.

[22] De Camargo MAF, Antunes JLF (2008). Untreated dental caries in children with cerebral palsy in the Brazilian context. Int J Paediatr Dent.

[23] Doneria D, Thakur S, Singhal P, Chauhan D, Jayam C, Uppal A (2017). Comparative evaluation of caries status in primary and permanent molars in 7–8-year-old schoolchildren of Shimla using caries assessment spectrum and treatment index. Contemp Clin Dent.

[24] Rosenbaum P, Paneth N, Leviton A, Goldstein M, Bax M, Damiano D (2007). A report: the definition and classification of cerebral palsy April 2006. Dev Med Child Neurol Suppl.

[25] Santos MT, Guare RO, Celiberti P, Siqueira WL (2009). Caries experience in individuals with cerebral palsy in relation to oromotor dysfunction and dietary consistency. Spec Care Dentist.

[26] Oredugba F (2011). Comparative oral health of children and adolescents with cerebral palsy and controls. J Disabil Oral Health.

[27] da Dourado MR, Andrade PMO, Ramos-Jorge ML, Moreira RN, Oliveira-Ferreira F (2013). Association between executive/attentional functions and caries in children with cerebral palsy. Res Dev Disabil.

[28] Lima DP, Diniz DG, Moimaz SAS, Sumida DH, Okamoto AC (2010). Saliva: reflection of the body. Int J Infect Dis.

[29] Santos MT, Ferreira MCD, Mendes FM, Oliveira GR (2014). Assessing salivary osmolality as a caries risk indicator in cerebral palsy children. Int J Paediatr Dent.

[30] Diniz MB, Guaré RO, Ferreira MCD, Santos MT (2015). Does the classification of cerebral palsy influence caries experience in children and adolescents?. Braz J Oral Sci.

[31] Santos MT, Nogueira M (2005). Infantile reflexes and their effects on dental caries and oral hygiene in cerebral palsy individuals. J Oral Rehabil.

[32] Tahmassebi JF, Curzon MEJ (2003). The cause of drooling in children with cerebral palsy—hypersalivation or swallowing defect?. Int J Paediatr Dent.

[33] Reid SM, Mccutcheon J, Reddihough DS, Johnson H (2012). Prevalence and predictors of drooling in 7-to 14-year-old children with cerebral palsy: a population study. Dev Med Child Neurol.

[34] Santos MT, Siqueira WL, Nicolau J (2007). Amylase and peroxidase activities and sialic acid concentration in saliva of adolescents with cerebral palsy. Quintessence Int.

[35] Subramaniam P, Babu KG, Rodriguez A (2010). Relation of salivary risk factors to dental caries in children with cerebral palsy. J Clin Pediatr Dent.

[36] Peluso I, Raguzzini A (2016). Salivary and urinary total antioxidant capacity as biomarkers of oxidative stress in humans. Pathol Res Int.

[37] Aycicek A, Iscan A (2006). Oxidative and antioxidative capacity in children with cerebral palsy. Brain Res Bull.

[38] Battino M, Ferreiro M, Gallardo I, Newman H, Bullon P (2002). The antioxidant capacity of saliva. J Clin Periodontol.

[39] Nicolas GG, Lavoie MC (2011). *Streptococcus mutans* and oral streptococci in dental plaque. Can J Microbiol.

[40] Carbonnelle E, Mesquita C, Bille E, Day N, Dauphin B, Beretti JL (2011). MALDI-TOF mass spectrometry tools for bacterial identification in clinical microbiology laboratory. Clin Biochem.

[41] dos Santos MTBR, Masiero D, Simionato MRL (2002). Risk factors for dental caries in children with cerebral palsy. Spec Care Dentist.

[42] Bensi C, Costacurta M, Docimo R (2020). Oral health in children with cerebral palsy: a systematic review and meta-analysis. Spec Care Dent.

