# Supplementary material for: Evaluation of salivary parameters and Streptococcus’ Mutans count in children with cerebral palsy in Egypt: a case control study
Source: BMC Oral Health. 2022 Sep 19;22:411. doi: 10.1186/s12903-022-02447-0 (PMC9487054; doi:10.1186/s12903-022-02447-0)
Supplement: Supplementary file 1 — Additional file 1. Questionnaire. [file 12903_2022_2447_MOESM1_ESM.docx]

**Questionnaire for children with cerebral palsy**

**استمارة استبيان لبحث الحالة الصحية للفم و الاسنان للأطفال المصابين بالشلل الدماغي**

السيد ولي أمر الطفل.................

تحيه طيبه و بعد,

هذا الأستبيان يهدف الي :تقييم الي تقييم إجمالي السعه المضادة للأكسدة ومستوى المكورات السبحية المتحورة في لعاب الاطفال المصابين بالشلل الدماغي و مدى العلاقه بالنخر السني و مقارنتهم بالأطفال الأصحاء. برجاء التعاون بالاجابة على جميع الاسئلة حتى نستطيع تقديم خدمة صحية افضل لطفلك, وشكرا.

**بيانات شخصية:**

- المدرسة/ المؤسسة : ...................................................
- اسم الطفل : .........................
- السن : ...............................
- النوع: ذكر  انثى 
- العنوان : ..............................
- رقم التليفون : ........................
- اسم الطبيب المعالج : .........................................
- عنوان الطبيب المعالج : ................................................

**بيانات خاصة بالاسرة:**

- المؤهل الدراسي الخاص بالاب: جامعى  متوسط  اعدادى/ ابتدائى  أمي 
- مهنة الأب :................................................................
- المؤهل الدراسي الخاص بالام: جامعى متوسط  اعدادى/ ابتدائى  أمي 

مهنة الأم :..................................................................

**بيانات خاصة بصحة الفم و الاسنان:**

- هل زارطفلك طبيب الأسنان خلال العام الأخير: لا  نعم 
- ما السبب من هذه الزياره : المتابعه و الفحص الدوري  للعلاج و تخفيف الألم 
- هل يغسل طفلك أسنانه: لا  نعم 
- كم مرة يغسل طفلك أسنانه: بعد كل وجبة  مرتان  مرة  أقل 
- هل يكون غسيل الأسنان تحت اشراف او بمساعدة ولي الأمر: لا  نعم 
- هل يعانى الطفل من مشكلات فى غسيل الاسنان: لا  نعم 
- هل يستعمل طفلك معجون الأسنان يحتوى على الفلوريد: لا  نعم 
- كم عدد المرات التي يتناول فيها الطفل الوجبات الخفيفة السكريه خلال اليوم :

مره أو أقل  مرتان أو أكثر

- ما هو قوام الغذاء الذي يتناوله طفلك : صلب شبه صلب سائل

**Questionnaire for healthy children**

**استمارة استبيان لبحث الحالة الصحية للفم و الاسنان للأطفال الأصحاء**

السيد ولي أمر الطفل.................

تحيه طيبه و بعد,

هذا الأستبيان يهدف الي تقييم إجمالي السعه المضادة للأكسدة ومستوى المكورات السبحية المتحورة في لعاب الاطفال المصابين بالشلل الدماغي و مدى العلاقه بالنخر السني و مقارنتهم بالأطفال الأصحاء**.** برجاء التعاون بالاجابة على جميع الاسئلة حتى نستطيع تقديم خدمة صحية افضل لطفلك , وشكرا.

**بيانات شخصية**

- المدرسة: ...................................................
- اسم الطفل : .........................
- السن : ...............................
- النوع: ذكر  انثى
- العنوان : ..............................
- رقم التليفون : .........................

**بيانات خاصة بالاسرة:**

- المؤهل الدراسي الخاص بالاب: جامعى  متوسط  اعدادى/ ابتدائى  أمي 
- مهنة الأب :................................................................
- المؤهل الدراسي الخاص بالام: جامعى متوسط  اعدادى/ ابتدائى  أمي 

مهنة الأم :..................................................................

**بيانات خاصة بصحة الفم و الاسنان:**

- هل زارطفلك طبيب الأسنان خلال العام الأخير: لا  نعم 
- ما السبب من هذه الزياره : المتابعه و الفحص الدوري  للعلاج و تخفيف الألم 
- هل يغسل طفلك أسنانه: لا  نعم 
- كم مرة يغسل طفلك أسنانه: بعد كل وجبة  مرتان  مرة  أقل 
- هل يكون غسيل الأسنان تحت اشراف او بمساعدة ولي الأمر: لا  نعم 
- هل يعانى الطفل من مشكلات فى غسيل الاسنان: لا  نعم 
- هل يستعمل طفلك معجون الأسنان يحتوى على الفلوريد: لا  نعم 
- كم عدد المرات التي يتناول فيها الطفل الوجبات الخفيفة السكريه خلال اليوم :

مره أو أقل  مرتان أو أكثر

- ما هو قوام الغذاء الذي يتناوله طفلك : صلب شبه صلب سائل

**Examination sheet**

**A. Examination sheet**

Serial: Date

| 1. **Personal Data**:  - Name: - Age: - Gender: | 1. **Medical history:**  \| **Child with CP** \| **Child without CP** \| \| --- \| --- \| \|  \|  \| |
| --- | --- | --- | --- | --- | --- |
| 1. **Clinical Type of CP**  - Spastic ( ) - Dyskinetic ( ) - Ataxic ( ) | 1. **Topographic Type of CP**  - Bilateral involvement: ( )   - Quadriplegia.  - Diplegia.  - Triplegia.   - Unilateral involvement: ( )   - Monoplegia  - Hemiplegia |
| 1. **Functional Type of CP:**  - Level I ( ) - Level II ( ) - Level III ( ) - Level IV ( ) - Level V ( ) | 1. **Epilepsy** Yes ( ) No ( ) 2. **Mental Retardation:**   Yes ( ) No ( )   1. **Associated disability:** 2. **Medication used** |


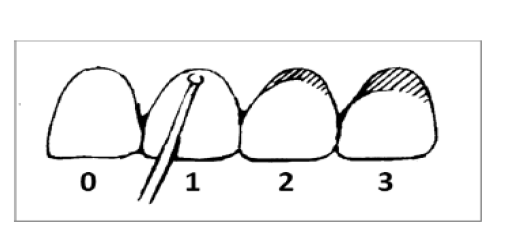


**B. Intra oral examination:**

***1-* Dental caries assessment:**

**Circle teeth numbers that correspond to those present in patient mouth**


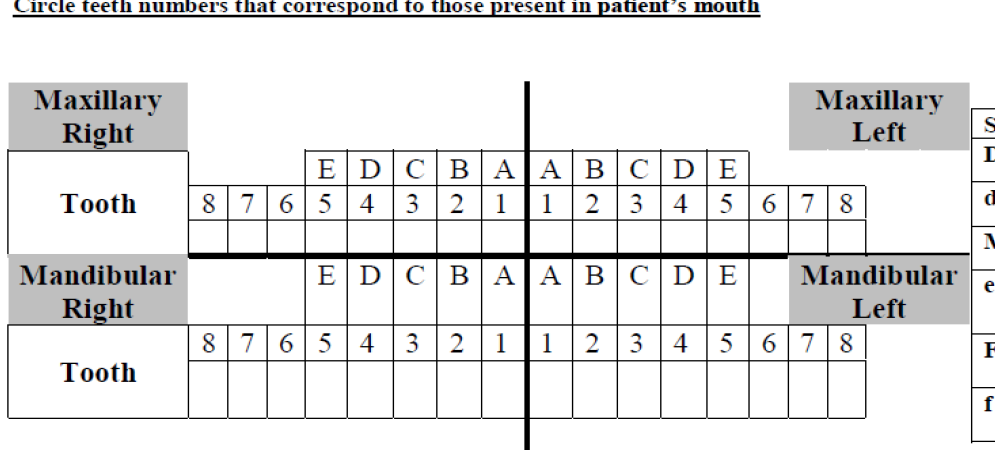


|  | **primary teeth** | **Permeant teeth** |
| --- | --- | --- |
| **total decayed teeth(d/D)** | **d =** | **D=** |
| **total filled teeth (f/F)** | **f =** | **F=** |
| **total missed teeth(m/M)** | **m =** | **M=** |
| **Total score** | **dmf=** | **DMF=** |

2- **OHI-S (**Greene and Vermillion**)**:


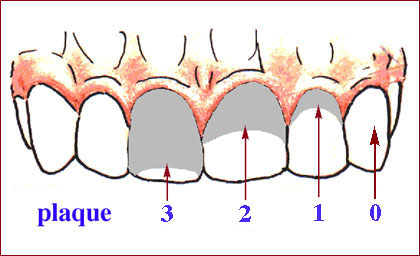


**Debris index**

|  | **Right molar** | | **Anterior** | | **Left molar** | | **Total** | |
| --- | --- | --- | --- | --- | --- | --- | --- | --- |
|  | Buccal | Lingual | Labial | Labial | Buccal | Lingual | Buccal | Lingual |
| Upper |  | - |  | - |  | - |  | - |
| Lower | - |  | - |  | - |  |  |  |

**Debris Index =** $\frac{(The buccal-scores) \mathbf{+} (The lingual-scores)}{Total number of examined buccal and lingual surfaces}$ =

**Calculus** **index**

|  | **Right molar** | | **Anterior** | | **Left molar** | | **Total** | |
| --- | --- | --- | --- | --- | --- | --- | --- | --- |
|  | Buccal | Lingual | Labial | Labial | Buccal | Lingual | Buccal | Lingual |
| Upper |  | - |  | - |  | - |  | - |
| Lower | - |  | - |  | - |  |  |  |

**Calculus Index =** $\frac{(The buccal scores) \mathbf{+} (The lingual scores)}{Total number of examined buccal and lingual surfaces}$ =

| **S-OHI for the patient** | = | **Debris Index + Calculus Index** |
| --- | --- | --- |

**C. Salivary Analysis:**

| **1- Drooling:**   - None  - Yes  | 2- **Salivary viscosity:**   - Watery and clear  - Thick, or sticky  |
| --- | --- |

1. **Salivary Flow rate**: (ml/min)
2. **Initial pH of saliva:**
3. **Salivary buffering capacity**: (ml acid/ ml saliva):
4. **Streptococcus mutans count**: (CFU/ ml )
5. **Salivary total antioxidant capacity**: (mmol/ L)
